# Seed germination and seedling development in response to submergence in tree species of the Central Amazonian floodplains

**DOI:** 10.1093/aobpla/plv041

**Published:** 2015-04-28

**Authors:** Risolandia Bezerra de Melo, Augusto César Franco, Clovis Oliveira Silva, Maria Teresa Fernandez Piedade, Cristiane Silva Ferreira

**Affiliations:** 1 Department of Botany, University of Brasilia, Brasilia, DF 70904-970, Brazil; 2 National Institute for Amazonian Research (INPA), INPA/Max-Planck Project, 69011-970, Manaus, Brazil

**Keywords:** Carbohydrate reserves, cell wall storage polysaccharides, flood tolerance, seed germination in water, submergence tolerance, tropism

## Abstract

Successful germination and seedling establishment are crucial steps for maintenance and expansion of plant populations and recovery from perturbations. Every year the Amazon River and its tributaries overflow and flood the adjacent forest, exerting a strong selective pressure on traits related to seedling recruitment. We examined seed characteristics, stored reserves, germination, seedling development and survival under water of eight representative tree species from the lower portions of the flood-level gradient to identify adaptive strategies that contribute to their regeneration in this extreme ecosystem. Submerged seedlings were assessed for longevity and survival until they showed symptoms of injury. At this point, the remaining healthy seedlings were planted in unsaturated soil to monitor recovery after re-exposure to air over 30 days. All small (seed mass ≤0.17 g) seeds had epigeal phanerocotylar-type germination, a trait that would allow plants to acquire light and CO_2_ in the shortest time. Cell wall storage polysaccharide was a major component of all seeds, suggesting plant investment in structural reserves. Seven of the eight species germinated and formed healthy seedlings under water that endured submersion without any apparent injury for periods of 20–115 days, depending on the species. Seedlings of some species changed the direction of root growth and grew towards the surface of the water, which might have increased the uptake of oxygen to the tissues. Only one of the seven species did not survive re-exposure to air. Species able to germinate and produce seedlings under submersion, which subsequently are able to establish in aerated soils, would have more time available for terrestrial growth. This is critical for colonization of lower portions of the flood-level gradient where establishment is constrained by the short terrestrial phase that precedes the next flood.

## Abstract (precis)

This study shows that Central Amazonian floodplain trees can cope with long-term flooding during the early life-history stages. Seven of the eight studied species germinated and formed seedlings under water that endured submersion without any apparent injury for periods of 20 to 115 days, depending on the species. Only one of the seven did not survive re-exposure to air. The ability to germinate and form seedlings in water that subsequently are able to thrive in aerated soils would allow the most use of the short terrestrial phase available for seedling establishment in the lower portions of the flood-level gradient.

## Introduction

Seedling recruitment is vital for persistence and expansion of plant populations and to ensure rapid recovery from perturbations. In seasonally flooded tropical forests, most species synchronize flowering and fruiting with the hydrological cycle and release their diaspores during the flood period, which favours dispersal by water or fish (Kubitzki and Ziburski 1994; Wittmann and Parolin 1999; Parolin 2002). On the other hand, seed dispersal in water could hinder germination because submergence may have negative effects on seed germination and seedling growth (Kozlowski 1997; Pérez-Ramos and Marañón 2009). Water can be damaging for germination because oxygen diffusion rates in water are ∼10^4^ times slower than those in air (Armstrong 1979). This slow rate is inadequate to support the vigorous respiration and other oxidative processes involved in germination by most species (Kozlowski and Pallardy 1997; Miro and Ismail 2013). Consequently, a large number of terrestrial species have high germination rates in unsaturated soils, but their seeds fail to germinate in water or saturated soils, and rapidly lose viability in these media due primarily to oxygen starvation (Parolin 2001).

Nevertheless, experiments have shown that seeds of certain tree species that are tolerant to flooding can remain viable even after long submergence periods (Lucas *et al.* 2012). Some can germinate or even form seedlings while they are still floating or submerged under a short column of water (Parolin and Junk 2002; Scarano *et al.* 2003; de Oliveira Wittmann *et al.* 2007; Ferreira *et al.* 2007). However, these studies did not accompany seedling survival and establishment after flooding. These processes are at risk from toxic products generated in anoxic soils and from reactive oxygen species (ROS) generated after flooding recedes and oxygen re-enters from the air (Ponnamperuma 1984; Blokhina *et al.* 2003; Miro and Ismail 2013). Oxidative stress resulting from ROS action can cause lipid peroxidation, necrosis of organs and even the death of the plant (Crawford *et al.* 1994; Blokhina *et al.* 2003; Colmer *et al.* 2014).

Every year, the River Amazon and its tributaries overflow and flood the adjacent forest, forming extensive wetlands. The Amazonian floodplains extend for more than 800 000 km^2^ and are the refuge for countless plant species, many of them endemic, which colonize the largest area of wetland forests in the world (Melack and Hess 2010; Wittmann *et al.* 2012). These forests are important because of their size and the variety of plant communities they house but also for their role in maintaining the hydrological cycle of the region and, consequently, the volume of freshwater discharged into the oceans by the Amazon River system (Junk *et al.* 1989; Melack and Hess 2010). The annual rise and fall of water plays a determining role in the early life-history stages of Amazonian wetland plants. Seed dispersal, germination and seedling establishment of most species are under strong selective pressure, brought about by this marked seasonal change in the water level.

In this study, we addressed the following questions: (i) Does the ability to germinate and form seedlings in water favour establishment in a seasonally flooded environment? Or, alternatively, would such events increase losses in the population after the flooding retreats? (ii) Could the germination and survival of these seedlings in water be related to the type of seed reserves? Accordingly, we examined seed characteristics, stored reserves, germination and seedling formation, under water, of eight tree species of the Amazonian wetland forests that are commonly present in the lower portions of the floodplain gradient and that can remain flooded for up to 230 consecutive days a year (Parolin *et al.* 2003; Parolin 2009; Ferreira *et al.* 2010; Wittmann *et al.* 2010). We also assessed the extent of their establishment in unsaturated soil after their removal from flooding.

## Methods

### Study areas, species and plant material collection

The Amazonian wetland forests are distributed along the major rivers and their tributaries. Every year these major rivers and their tributaries overflow and invade large areas of adjacent forests, regionally called várzea (flooded by white-water rivers) or igapó (flooded by black- or clear-water rivers) forests (*sensu* Prance 1979). The lower portions (low várzea and low igapó) of the flood-level gradient are environments subjected to extreme flooding conditions. The vegetation colonizing these areas is the first to be flooded and the last to be drained. It is characterized by a relatively low diversity of tree species; however, a relatively large number of them are endemic to this type of environment (Worbes 1997; Ferreira and Prance 1998; Piedade *et al.* 2005; Wittmann *et al.* 2012).

The eight species used in the present study are typical trees from the lower levels of the Amazonian floodplains and belong to eight different families (Table 1). Four species were collected in the low-várzea forests: *Crataeva tapia* (Capparaceae), *Laetia corymbulosa* (Salicaceae), *Pouteria glomerata* (Sapotaceae), *Pseudobombax munguba* (Malvaceae); and four species were collected in low-igapó forests: *Genipa americana* (Rubiaceae), *Eugenia inundata* (Myrtaceae), *Parkia discolor* (Fabaceae) and *Simaba guianensis* (Simaroubaceae).

**Table 1.** Seed dry mass (mean ± standard error; *n* = 50), dispersal syndrome, germination type and typical habitat of occurrence for eight tree species of Central Amazonian floodplains. Anemoc, anemochory; hydr, hydrochory; ichthy, ichthyochory; orni, ornithochory; zooc, zoochory; E, epigeal; H, hypogeal; cryp, cryptocotylar; phan, phanerocotylar; LI, low-igapó forests; LV, low-várzea forests.

Species	Dry mass (g)	Dispersal	Germination	Habitat
*Crataeva tapia*	0.17 ± 0.01	hydr, ichthy	E, phan	LV
*Eugenia inundata*	0.06 ± 0.01	ichthy	E, phan	LI
*Genipa americana*	0.05 ± 0.00	hydr, ichthy, zooc	E, phan	LI
*Laetia corymbulosa*	0.01 ± 0.01	ichthy, orni	E, phan	LV
*Parkia discolor*	0.34 ± 0.02	hydr, zooc	E, phan	LI
*Pouteria glomerata*	2.31 ± 0.09	hydr, ichthy, zooc	H, cryp	LV
*Pseudobombax munguba*	0.07 ± 0.00	anemoc, hydr	E, phan	LV
*Simaba guianensis*	0.21 ± 0.03	ichthy	H, cryp	LI

The samples were collected between May and June 2011, when one of the largest floods was recorded for these rivers. This flood lasted 246 days (CPRM 2011) and the average height of the water column in the sampled areas was 5 m. Ripe fruits were collected from each species (three to five individuals, according to availability) in the lower levels of the flood terrain (low várzea or low igapó). Fruits from the low-várzea forests were collected from Marchantaria Island (3°1′S; 60°9′W) and in the surrounding areas at the margins of the Solimões River. Fruits from the low-igapó forests were collected along tributaries of the Negro River, in the Sustainable Development Reserve of Tupé (3°2′S; 60°15′W) and at the margins of the Tarumã-Mirim River (3°14′S; 59°57′W). All sites were located ∼20 km from the city of Manaus, Brazil. After collection (Registration for collection SISBIO 10257-1, Ministry of Environment, Water Resources and Legal Amazon, Brazil), fruits were stored and transported in thermal boxes to the Laboratory of Plant Physiology of the University of Brasilia (UNB) in the city of Brasília, Brazil, where seeds were extracted from the fruits and thoroughly mixed together in a single lot to obtain compound samples.

### Seed characteristics

In this study, we use the term ‘seed’ in the strict botanical sense to define the ovule developed after fertilization that contains an embryo (embryonic axis and cotyledons) and reserve tissue (sometimes absent), both being protected by a seed coat (integument) (Beltrati and Paoli 2003; Fenner 2004).

Seed sizing was based on dry mass, according to the International Rules for Seed Testing [International Seed Testing Association (ISTA) 2014]. A lot consisting of 50 seeds of each species was dried at 105 ± 3 °C for 24 h. The seeds were then weighed on a 0.0001 g precision scale. Information on the dispersal syndromes of the species was obtained from the data available in the literature (Kubitzki and Ziburski 1994; Parolin *et al.* 2013).

### Biochemical analyses in whole seeds

For the analysis of reserves, the seeds were lyophilized for 48 h, weighed to determine dry mass and subsequently ground to powder in a ball mill (Tecnal Ltda). Seeds were analysed for protein, lipids, non-structural carbohydrates (starch, contents and composition of soluble sugars) and cell wall storage polysaccharides (CWSPs). Total proteins were quantified by the Bradford method (1976) and total lipids were quantified by adapting the methods proposed by Ramadan *et al.* (2009) and Metherel *et al.* (2009), where a 200 mg sample was subjected to three extractions with 2 mL of hexane by ultrasound for 25 min, followed by collection of the supernatant. The resulting pellet was air dried in a laminar flow hood for 48 h to evaporate the solvent. The total amount of lipids was estimated by using a precision balance sensitive to 0.0001 g.

Starch was analysed by the enzymatic method as described by do Amaral *et al.* (2007), using a glucose standard curve. Soluble sugars were extracted in 80 % ethanol by the phenol–sulfuric acid method of Dubois *et al.* (1956). The composition of sugars was determined by high-performance anion exchange chromatography with a pulsed amperometric detector (HPAEC/PAD model DX500) and a CarboPak PA-1 column (Dionex Corporation, Sunnyvale, CA, USA) eluted with water, followed by a post-column reaction using flows of 1 mL min^−1^: 0–15 min, 200 mM NaOH (50 %) and H_2_O (50 %); 15–20 min, 200 mM NaOH (100 %); 20–25 min, 200 mM NaOH (50 %) and H_2_O (50 %). The areas of each peak were corrected in accordance with the sensitivity of the detector for each sugar (Santos and Buckeridge 2004).

Fractionation of cell walls followed the methodology of Gorshkova *et al.* (1996), which involves the extraction of soluble sugars and starch from a 300 mg sample. Analysis of CWSP monosaccharide composition after acid hydrolysis was performed by HPAEC/PAD as described above.

Calibration curves were fitted by regression analysis to determine the concentrations of soluble sugar, starch, carbohydrates, proteins and CWSPs present in the whole seed (*r*^2^ = 0.99). The content of each of these compounds was calculated in relation to the dry mass of the seed.

### Germination and seedling development under submergence, and survival after re-exposure to air

For simulation of flooding events, a completely randomized design with two treatments, four replicates and eight species was used. Treatments were: submerged (seeds submerged in 300 mL of distilled water; water column of ∼3 cm) and control (non-submerged, with substrate consisting of double filter paper kept moist). In both treatments, seeds were sealed in plastic gearbox-type boxes (11 × 11 × 3.5 cm; four replicates of 25 seeds each, except for *P. glomerata* in which four replicates of 15 seeds each were used), and kept in a B.O.D-type germination chamber (model MA402/1, Marconi^®^, Brazil) at 28 °C, with a 12-h photoperiod and photosynthetic photon flux density of 30 µmol m^−2^ s^−1^ delivered by white fluorescent lamps. *Parkia discolor* was the only species requiring a scarification pretreatment for germination. Germination occurred with the emergence and curvature of the radicle (∼1 cm). Percentage germination (%), mean germination time (MGT) and the type of germination were recorded. The mean germination time and percentage germination were calculated in accordance with the formulae described by Labouriau (1983). The viability of non-germinated seeds at the end of the experiment was assessed by the tetrazolium test (Moore 1973).

Seeds that germinated in water were transferred to transparent plastic pots containing 500 mL of distilled water that were kept closed (*n* = 4 pots per species), while the controls were transferred to commercial Bioplant^®^ soil. A seedling was considered to be formed when it had a well-developed root and shoot system (presence of caulicle and cotyledonary leaves or first pair of leaves). Submerged seedlings were regularly assessed for longevity and survival until they showed symptoms of injuries in the form of root apex necrosis. When this symptom first appeared in two to three seedlings of each species, the remaining healthy-looking seedlings were transferred to commercial Bioplant^®^ substrate soil and recovery and survival were monitored for 30 days. Seedlings were maintained under the same conditions of photoperiod, light and temperature used during germination.

The concentration of oxygen dissolved in the water was measured weekly in the submergence treatment during the whole duration of the experiment with a digital oximeter (DM-4P Digmed), resulting in a mean (± standard deviation) of 4.9 (± 1.0) mg L^−1^ at 27 °C, corresponding to 62 % saturation. This is within the range measured in surface waters of the Amazon River (4.0–5.5 mg L^−1^; Furch and Junk 1997).

### Statistical analysis

The package lme4 (Bates *et al.* 2013) was used for linear mixed model analyses for a randomized block design to test for effects of species and treatments (submerged and non-submerged) on germination, MGT and successful seedling development (Quinn and Keough 2002). The mean germination time was log_10_-transformed before the analysis. Type III analysis of variance (ANOVA) tables and *t*-tests based on Satterthwaite's approximations were obtained using the package lmerTest (Kuznetsova *et al.* 2013). Restricted maximum likelihood (REML) estimates of the parameters in linear mixed-effects models were determined using the lmer function in the lme4 package. All analyses were performed using the software R v. 3.1 (R Development Core Team 2008). For all tests, differences were considered significant at *P* < 0.05.

## Results

### Seed characteristics and germination type

Five of the eight studied species (62.5 %) had small seeds, with seed mass ranging from 0.01 to 0.17 g (Table 1), and germination of epigeal-type phanerocotylar. The remaining three species (*P. glomerata*, *S. guianensis* and *P. discolor*) had large seeds with mass between 0.21 and 2.31 g, and cryptocotylar hypogeal (*P. glomerata* and *S. guianensis*) or epigeal (*P. discolor*)-type germination. All species can be dispersed by water (hydrochory) and/or fish (ichthyochory) after the seeds fall into the water.

### Biochemical analysis of whole seeds

Analysis of the major storage compounds in the seeds showed that species preferentially accumulate cell wall polysaccharides (high packing density) in amounts that ranged from 22 to 52 % of seed dry mass (data not shown; can be calculated using the data from Table 2). Of the eight studied species, seven had much more CWSP than the other types of storage compounds (Table 2). The exception was *C. tapia*, which showed similar quantities of lipids and CWSPs. The protein content was low for all species with the highest percentage being found in *P. glomerata* seeds. The content of total soluble sugars (TSS) and starch varied substantially among species with *G. americana* showing the highest TSS concentrations and *L. corymbulosa* showing the lowest. Starch reserves were highest in *P. glomerata* and *E. inundata*, and lowest in *P. discolor* and *P. munguba*.

**Table 2.** Major storage reserves expressed as a percentage of the total amount of reserves in seeds of eight tree species of Central Amazonian floodplains. TSS, total soluble sugars; CWSP, cell wall storage polysaccharide; Total, the total amount of reserves expressed as a percentage of seed dry mass.

Species	TSS	Starch	Protein	Lipids	CWSP	Total
*Crataeva tapia*	4.9	22.0	1.3	37.7	34.1	94.6
*Eugenia inundata*	17.2	25.0	0.5	1.6	55.7	68.8
*Genipa americana*	25.0	1.9	2.0	15.9	55.2	39.3
*Laetia corymbulosa*	4.0	4.2	0.9	43.9	47.0	79.3
*Parkia discolor*	10.4	0.3	1.1	10.8	77.4	66.6
*Pouteria glomerata*	6.2	25.2	3.0	9.4	56.2	64.8
*Pseudobombax munguba*	8.2	0.3	1.3	40.8	49.4	99.0
*Simaba guianensis*	8.7	13.6	1.3	30.8	45.6	92.1

Among the soluble sugars, sucrose predominated in all species except *C. tapia*, where 80 % of the TSS comprised glucose and fructose (Table 3). Although sucrose was the main sugar in *G. americana*, *P. discolor*, *P. glomerata* and *S. guianensis*, seeds of these species also contained substantial amounts of fructose, glucose or raffinose (Table 3).

**Table 3.** Composition (%) of soluble sugars in seeds of eight tree species of Central Amazonian floodplains.

Species	Soluble sugar (%)			
	Fructose	Glucose	Raffinose	Sucrose
*Crataeva tapia*	30.9	49.1	0.8	19.2
*Eugenia inundata*	4.2	9.1	4.2	82.5
*Genipa americana*	24.4	13.7	5.3	56.6
*Laetia corymbulosa*	2.1	3.3	10	84.6
*Parkia discolor*	19.2	0.1	22.5	58.2
*Pouteria glomerata*	13.3	22.4	7.4	56.9
*Pseudobombax munguba*	0.6	9.7	9.1	80.6
*Simaba guianensis*	1.7	29.1	26.4	42.8

Acid hydrolysis revealed remarkable differences between the species in the chemical structure of CWSPs, as defined by the proportion of the different monosaccharides (Table 4; Buckeridge *et al.* 2000*b*). Galactose and mannose were the most abundant monosaccharides in *L. corymbulosa* and *P. discolor* seeds and they were found in ratios higher than 3 : 1, suggesting the presence of a galactomannan. In *P. munguba*, *E. inundata*, *S. guianensis* and *C. tapia* seeds, high proportions of glucose, as well as galactose and mannose, suggest a galactoglucomannan. Reserve polysaccharides of *P. glomerata* and *G. americana* seeds were composed mostly of arabinose (>40 %) and galactose (>30 %), with proportions that correspond to that of an arabinogalactan (Table 4).

**Table 4.** Monosaccharide analysis after acid hydrolysis of CWSPs in seeds of eight tree species of Central Amazonian floodplains. Composition of monosaccharides expressed as a percentage of total corrected monosaccharide peak area as determined by high-performance liquid chromatography.

Species	Monosaccharides (%)				
	Arabinose	Galactose	Glucose	Manose	Xylose
*Crataeva tapia*	0.0	11.4	58.7	30.0	0.0
*Eugenia inundata*	0.0	28.2	32.9	38.1	0.9
*Genipa americana*	49.1	39.1	3.4	6.9	1.5
*Laetia corymbulosa*	0.0	70.6	4.0	16.5	4.8
*Parkia discolor*	2.7	68.2	1.8	20.3	7.0
*Pouteria glomerata*	57.6	39.0	1.8	0.6	0.9
*Pseudobombax munguba*	0.0	39.8	17.3	36.2	6.6
*Simaba guianensis*	0.0	60.9	13.9	24.3	0.9

### Seed germination in submerged conditions

The results of the ANOVA revealed that the interaction submersion × species was significant for seed germination (Table 5). Seven of the eight studied species successfully germinated in water (Table 6). The exception was *P. discolor*. The seeds from this species, although able to take up water, subsequently fermented and necrotized in a few days under submergence. However, the species showed high germination under control conditions (Table 6). Submersion did not affect the germination percentage of four species (*C. tapia*, *G. americana*, *L. corymbulosa* and *P. glomerata*) but significantly (*P* < 0.05) decreased the germination of *S. guianensis* and *P. munguba*. On the other hand, more seeds of *E. inundata* (*P* < 0.05) germinated when submerged, compared with controls (Table 6).

**Table 5.** Results of the Type III ANOVA on the effect of submersion, species and submersion and species interaction on seed germination, successful seedling development and MGT.

Response variable	Source of variation	df	*F*-value	*P*
Seed germination	Treatments	1	35.66	<0.001
	Species	7	34.43	<0.001
	Treatments × species	7	15.16	<0.001
	Residual	45		
Sucessful seedling development	Treatments	1	41.48	<0.001
	Species	7	26.29	<0.001
	Treatments × species	6	40.15	<0.001
	Residual	42		
MGT	Treatments	1	31.50	<0.001
	Species	7	312.09	<0.001
	Treatments × species	6	10.36	<0.001
	Residual	42

**Table 6.** Effect of submersion on the percentage germination (number of germinating seeds in relation to the total number of seeds) and MGT for seeds of eight tree species of Central Amazonian floodplains. Non-submerged seeds were placed on moistened double-layered filter paper. Submerged seeds were kept in 300 mL distilled water (water column of ∼3 cm). In both treatments, four replicates of 25 seeds each were used, except *P. glomerata* in which four replicates of 15 seeds each were used. Data expressed as mean ± standard error. Bold values indicate that non-submerged and submerged seeds differed significantly (*P* < 0.05) according to the REML test.

Species	Germination (%)		*P*	MGT (days)		*P*
	Non-submerged	Submerged		Non-submerged	Submerged	
*Crataeva tapia*	97.0 ± 1.0	89.0 ± 1.0	0.600	8.4 ± 0.2	12.7 ± 0.4	**0**.**014**
*Eugenia inundata*	59.0 ± 4.4	88.0 ± 5.2	**0**.**008**	10.3 ± 1.0	9.7 ± 1.4	0.112
*Genipa americana*	29.0 ± 10.6	23.0 ± 5.7	0.882	21.6 ± 3.9	21.5 ± 2.1	0.130
*Laetia corymbulosa*	64.0 ± 7.5	66.0 ± 6.6	0.459	58.7 ± 3.6	72.8 ± 4.7	0.322
*Parkia discolor*	88.0 ± 5.4	0.0	**<0**.**001**	5.2 ± 0.0	Not germinated	NA
*Pouteria glomerata*	15.0 ± 6.9	13.3 ± 8.2	0.604	188.5 ± 9.3	154.5 ± 8.1	**0**.**002**
*Pseudobombax munguba*	89.0 ± 5.3	54.0 ± 18.5	**0**.**049**	2.2 ± 0.1	7.1 ± 1.2	**<0**.**001**
*Simaba guianensis*	93.0 ± 1.0	40.0 ± 5.2	**0**.**002**	59.8 ± 4.8	110.8 ± 4.1	**<0**.**001**

The tetrazolium test for viability was performed on seeds of *C. tapia*, *E. inundata*, *G. americana*, *P. discolor* and *S. guianensis* that did not germinate in the two treatments (submerged and control). The difficulty in identifying and/or handling embryos of *L. corymbulosa*, *P. munguba* and *P. glomerata* made it impossible to perform the tetrazolium test in seeds from these species. The percentage of potentially viable seeds was obtained from the total number of seeds that did not germinate. The proportion of viable embryos among seeds that did not germinate when under water was 27.3 % for *C. tapia*, 2.6 % for *G. americana* and 37.5 % for *S. guianensis*. All (100 %) *E. inundata* and *P. discolor* seeds that did not germinate under water were non-viable. Among the control seeds that did not germinate, only *G. americana* retained some viable embryos (10.2 %). The remaining species had only seeds with non-viable embryos (100 %).

The species × submersion interaction term of the ANOVA was significant for the MGT (Table 5). Submersion increased the MGT of three species (*C. tapia*, *P. munguba* and *S. guianensis*) and decreased the MGT of *P. glomerata. Pouteria glomerata* was also the species with the longest MGT.

### Seedling development in the water, longevity under submergence conditions and survival after flooding

**Figure 1.**
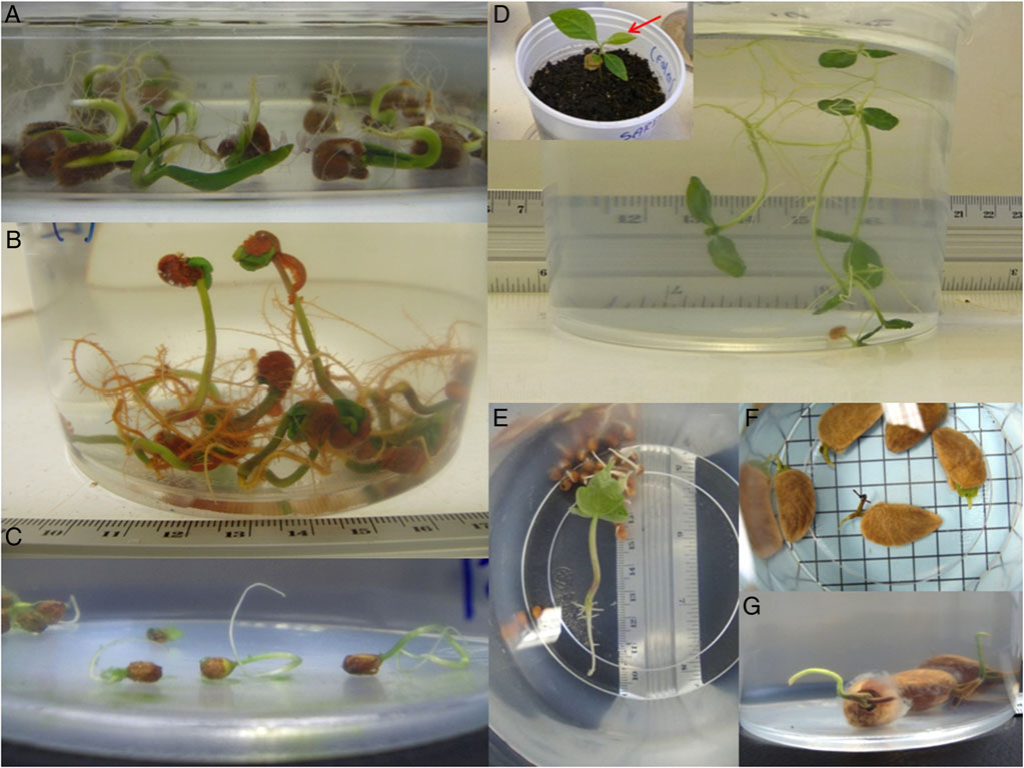
Germinated seeds and seedlings formed in water. (A) *Crataeva tapia*; (B) *Eugenia inundata*; (C) *Laetia corymbulosa*; (D) *Genipa americana*; (E) *Pseudobombax munguba*; (F) *Simaba guianensis*; (G) *Pouteria glomerata*. The arrow in the inset of (D) points to new leaves that flushed after the seedling was removed from water following a period of 90 days of submersion and transplanted into aerated soil.

**Figure 2.**
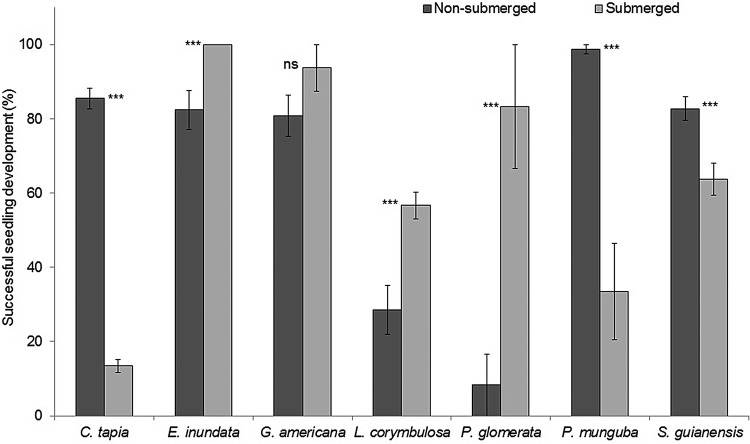
Successful seedling development in submerged (kept submerged in distilled water) and non-submerged (grown in commercial Bioplant^®^ substrate soil) conditions expressed as a percentage of the number of germinated seeds for seven tree species of Central Amazonian floodplains. The asterisks indicate significant differences between the two treatments according to the REML test; ***P < 0.001. Bars are means ± standard errors.

All seven species in which seeds germinated in water formed seedlings when submerged (Figs 1 and 2). The highest percentages of seedling formation during submergence were observed in *E. inundata* (100 %), followed by *G. americana* (93.8 %), *P. glomerata* (83.3 %) and *S. guianesis* (63.7 %). However, the submersion × species effect was significant (Table 5). Thus, seedling formation in *E. inundata*, *L. corymbulosa* and *P. glomerata* was enhanced by submersion and not affected in *G. americana*. In contrast, submersion significantly (*P* < 0.05) reduced the production of seedlings in *C. tapia*, *P. munguba* and *S. guianesis* (Fig. 2).

Seedlings that developed in water could remain submerged without showing any apparent signs of injury for a period ranging from ∼3 weeks to almost 4 months, depending on the species. Roots of some species showed a change in the direction of growth and grew towards the water surface (Fig. 1A and D). The species that showed least tolerance to submergence was *P. munguba* with the first signs of root necrosis appearing 20 days after germination (Table 7). All other species remained healthy looking (green and with white roots) in the water for a period of ∼2 months (*P. glomerata*) to close to 4 months (*E. inundata*).

**Table 7.** Seedling longevity in water and percent survival after being transplanted into unsaturated soil for seven tree species of Central Amazonian floodplains. Longevity is the number of days that seedlings were kept submerged in pots with 500 mL distilled water until 2–3 seedlings of the species showed root apex necrosis. After the first signs of injuries were detected, the remaining healthy-looking seedlings were removed from the water, transplanted into unsaturated soil and, after 30 days, the number of surviving seedlings was determined as a percentage of the initial number of transplanted seedlings.

Species	Longevity (days)	Seedling survival in soil (%)
*Crataeva tapia*	90	70
*Eugenia inundata*	115	93
*Genipa americana*	90	80
*Laetia corymbulosa*	100	13
*Pouteria glomerata*	59	0
*Pseudobombax munguba*	20	85
*Simaba guianensis*	77	80

After the first signs of injury were detected, seedlings were removed from the water and transplanted into unsaturated soil (inset in Fig. 1D). Seedlings of *P. glomerata* did not survive after they were removed from the water and transplanted into unsaturated soil, and seedlings of *L. corymbulosa* had low survival. All other species had high survival after 30 days of being transplanted into soil (Table 7).

## Discussion

In the flooded areas of the Amazon, it has been postulated that large seeds would be found mostly in nutrient-poor environments such as igapó forests, and that small seeds would be prevalent in nutrient-rich várzea forests (Parolin 2000; Parolin *et al.* 2010). In the current study, we observed a marked heterogeneity in seed size regardless of whether the species was from low-várzea or low-igapó areas. However, all small (seed mass ≤0.17 g) seeds had epigeal phanerocotylar-type germination, which can be considered of adaptive advantage in helping them to acquire light and CO_2_ in the shortest time, thus ensuring onward development of the plant (Kitajima 2002; Baraloto and Forget 2007). This is especially relevant for the flooded areas of the Amazon, where flooding leaves only a short period of time for the seedlings to establish.

On the other hand, there was a convergence with respect to the main type of stored reserves. All eight species preferentially accumulated CWSPs and lipids, which are considered compacted reserves with a high energetic value. The presence of small fractions of xylose possibly arose from the persistence of seed coat residues (integument) during seed processing for CWSP analysis. The protein content stored in the seed was low (mean value 1.5 %) and similar to those reported by Parolin *et al.* (2010) in a review study on Amazonian species belonging to 20 different families. Although low protein values are common in seeds of native species (Baú *et al.* 2001; da Silva Ferreira *et al.* 2009), these compounds are key sources of nitrogen and sulfur for the embryo and are needed for nucleic acid and amino acid synthesis, and thus for the synthesis of new proteins and enzymes (Shewry *et al.* 1995; Buckeridge *et al.* 2004). In the case of seeds of flooded plants, the reserve proteins may also contribute to the synthesis of adaptive anaerobic metabolic enzymes, since most of the species we studied germinated and produced seedlings under water.

The CWSP content found in seeds of the eight species (22–52 % of seed dry mass) is relatively high. Most species that typically accumulate CWSP in seeds, such as tropical legumes (Fabaceae), show values that do not exceed 25 % of seed dry mass (Buckeridge *et al.* 2000*a*). Species such as *Mimosa acustistipula* and *Hymenaea courbaril* possess a CWSP of ∼40 % of seed dry mass (Buckeridge *et al.* 2000*a*; Santos and Buckeridge 2004) while CWSP values >50 % have been reported for only a few species, e.g. *Coffea arabica* (Rubiaceae) (Joët *et al.* 2014) and *Himatanthus sucuuba* (Apocynaceae) (da Silva Ferreira *et al.* 2009). The CWSP reserves are mobilized after germination and play an important role in providing carbon and energy for seedling construction (Buckeridge *et al*. 2000*b*), a function also carried out by reserve lipids (Levin 1974).

The species we investigated are typical of the lower portions of the flood-level gradient (low várzea and low igapó). This environment is subjected to extreme disturbance. During the critical stages of establishment of a new plant, the importance of reserves accumulated by the seed becomes clearer. In addition to being substrate reserves, galactomannan-, galactoglucomannan- and arabinogalactan-type CWSPs can have other functions in the seed, such as imbibition and radicle protrusion control (for a review, see Buckeridge *et al*. 2000*a*; Buckeridge 2010). For the eight species of the study, such versatility in function may be an important attribute, since, in the Amazonian flood areas, seeds are released in the flood period (hydrochoric or ichthyochoric dispersal). The delay observed in the germination of three of the seven species that germinated in the water could be the result of the CWSPs’ mediation in controlling seed imbibition and reserve mobilization to the embryo. Studies are needed to unravel the role of these compounds during germination and find out how they are utilized in maintaining seedling metabolism and development under water, as well as their role in prolonging seedling longevity under such conditions.

Among the eight species studied, seven germinated and formed seedlings in water that, depending on the species, were able to withstand 20–115 days of submergence without showing signs of root tip necrosis. Some species were also able to change the direction of root growth and grew towards the surface of the water, which might have increased the uptake of oxygen to the tissues. Experiments on the response of seedlings of *Arabidopsis* and tomato to salinity gradients in soil (Pierik and Testerink 2014) showed that they are capable of changing the direction of growth of their roots to ‘escape’ or reduce their exposure to salinity (negative halotropism). Although further studies are necessary, modulation of the direction of root growth to escape low-O_2_ stress may also be important to submergence tolerance.

Seedlings of *P. munguba* were the most sensitive to submergence. Although this species is a low-várzea pioneer (Wittmann *et al.* 2010), seedlings of this species developed in water were unable to withstand more than 20 days of submergence without showing signs of root tip necrosis. Furthermore, ∼70 % of germinated seeds died before developing into seedlings. On the other hand, it exhibits ecological strategies that prevent the seeds from being exposed to long periods of submergence. These include the timing of fruit dispersal to occur at the peak of the flooding period, and the presence of hairs that enclose the seed and facilitate dispersal by wind/water and allow the seed to float (Kubitzki and Ziburski 1994; Ferreira *et al.* 2010). Additionally, epigeal phanerocotylar germination and fast early growth would ensure that the species is established before the next flood.

Although there are reports in the literature that some Amazonian floodplain species can germinate in water (de Oliveira Wittmann *et al.* 2007; Ferreira *et al.* 2007; our results), this strategy is not common among terrestrial trees. Even when it can take place, it does not in itself lead to successful seedling establishment in seasonally flooded ecosystems. Our study of the species occurring in the Amazonian wetland forests seems to be the first to monitor not only germination and seedling development in the water but also the ability to survive under these conditions and to establish after the flood period. For instance, submersion did not affect germination of *G. americana* and *L. corymbulosa* or prevent germinated seeds of these two species from developing into seedlings. In fact, submersion had a positive effect on seedling production of *L. corymbulosa*. However, most seedlings of this species died when they were removed from the flood conditions and transplanted into unsaturated soil. Re-aeration has been considered to be more problematic than the low levels of oxygen imposed by flooding *per se* (Biemelt *et al.* 1998; Miro and Ismail 2013). According to Lopez and Kursar (1999), the way plants respond to post-flooding events (oxygen toxicity and soil drying) plays an important role in determining which species are most competitive in seasonally flooded environments. Therefore, the ability to germinate in water is not a guarantee of success in seedling establishment after the flood waters recede.

*Eugenia inundata* was the only species to have its germination percentage enhanced under flooding conditions. In addition, all seeds that germinated under submergence not only formed viable seedlings but also showed the highest tolerance to submergence. The first signs of root tip necrosis appeared only after 115 days of submergence. Seedlings of this species also had the greatest survivorship when they were removed from the water and planted in the soil. In igapó forests, the family Myrtaceae is among those with the largest number of species (Scudeller and Souza 2009). *Eugenia inundata* is one of the dominant species, especially in the lower flooding areas, where plants are flooded most of the year (Takeuchi 1962). In contrast, of the species that were able to germinate in water, *P. glomerata* was the one with the lowest germination percentages, both under flooded and non-flooded conditions. Germination occurred slowly and erratically over a long period. This germination behaviour is indicative of physiological dormancy with immaturity of the embryo being a likely cause (Baskin and Baskin 1998). However, due to the hardness of the seed coat, it was not possible to analyse the embryo and to test the viability of the seeds that did not germinate. Although seedling formation occurred in the water, they were unable to survive after transfer to aerated soil, which suggests that the ability to colonize wetland areas appears to be related to dormancy mechanisms that prevent or delay their germination in water. The dormancy mechanisms in *P. glomerata* and how they relate to the optimal conditions for seed germination need further investigation (Cruz 2005).

*Parkia discolor* was unable to germinate under water. However, the indehiscent pods of *P. discolor* can float and the presence of a rigid and impermeable seed coat prevents seed imbibition, delaying germination and maintaining seed viability even after prolonged submergence (Scarano 1998). Scarified seeds of *P. discolor* germinated rapidly in well-aerated soil. There are scant references in the literature to bat dispersal and the attractiveness of the fruits of this species to monkeys and parrots (Hopkins 1986). Information on seed dispersers of this species is critical to better assess strategies favouring seed germination and seedling establishment of this species in these seasonally flooded ecosystems.

## Conclusions

This study shows that Central Amazonian floodplain trees have different mechanisms to cope with long-term flooding during the early life-history stages. For *E. inundata*, *G. americana* and *S. guianensis*, the ability to germinate and form seedlings in water, their tolerance to long-term submergence and high survival after being transplanted into aerated soil can favour a fast establishment of the young individual during the period of receding water. In contrast, the timing of fruit dispersal and dispersal syndrome might be critical for species such as *P. discolor*, which has a rigid and impermeable seed coat and is not able to successfully germinate in water, or *P. munguba*, in which seedlings are not as tolerant to long-term submergence. Further work is needed on the regulatory mechanisms behind the mobilization and depletion of seed reserves during germination and in maintaining seedling metabolism and development under flooded conditions, as well as in prolonging seedling longevity under such conditions.

## Sources of Funding

Funding for this work was provided by Programa Nacional de Apoio e Desenvolvimento da Botânica from Coordenação de Aperfeiçoamento de Pessoal de Nível Superior (Brazil) and by the Universal Programme from Conselho Nacional de Desenvolvimento Científico e Tecnológico (Brazil) to A.C.F., C.S.F. and M.T.F.P. CAPES (Brazil) provided a graduate scholarship to R.B.d.M.

## Contributions by the Authors

A.C.F. and C.S.F. designed the study. C.S.F., R.B.d.M. and C.O.S. established the biochemical protocols. R.B.d.M. performed the experiments and collected the data (with assistance from C.S.F. and C.O.S.). C.S.F., R.B.d.M. and A.C.F. analysed and interpreted the data. A.C.F., C.S.F. and M.T.F.P. wrote the paper.

## Conflict of Interest Statement

None declared.
